# Three-Device (3D) Technique for Liver Parenchyma Dissection in Robotic Liver Surgery

**DOI:** 10.3390/jcm10225265

**Published:** 2021-11-12

**Authors:** Aristotelis Perrakis, Mirhasan Rahimli, Andrew A. Gumbs, Victor Negrini, Mihailo Andric, Jessica Stockheim, Cora Wex, Eric Lorenz, Joerg Arend, Mareike Franz, Roland S. Croner

**Affiliations:** 1 University Clinic for General, Visceral, Vascular and Transplant Surgery, University of Magdeburg, Leipzigerstr. 44, 39120 Magdeburg, Germany; mirhasan.rahimli@med.ovgu.de (M.R.); victor.negrini@med.ovgu.de (V.N.); mihailo.andric@med.ovgu.de (M.A.); jessica.stockheim@med.ovgu.de (J.S.); cora.wex@med.ovgu.de (C.W.); eric.lorenz@med.ovgu.de (E.L.); joerg.arend@med.ovgu.de (J.A.); mareike.franz@med.ovgu.de (M.F.); roland.croner@med.ovgu.de (R.S.C.); 2 Department of Surgery, Centre Hospitalier Intercommunal de Poissy/Saint-Germain-en-Laye, 10 Rue du Champ Gaillard, 78300 Poissy, France; aagumbs@gmail.com

**Keywords:** robotic surgery, hepatectomy, liver metastasis, hepatocellular carcinoma, liver surgery

## Abstract

Background: The implementation of robotics in liver surgery offers several advantages compared to conventional open and laparoscopic techniques. One major advantage is the enhanced degree of freedom at the tip of the robotic tools compared to laparoscopic instruments. This enables excellent vessel control during inflow and outflow dissection of the liver. Parenchymal transection remains the most challenging part during robotic liver resection because currently available robotic instruments for parenchymal transection have several limitations and there is no standardized technique as of yet. We established a new strategy and share our experience. Methods: We present a novel technique for the transection of liver parenchyma during robotic surgery, using three devices (3D) simultaneously: monopolar scissors and bipolar Maryland forceps of the robot and laparoscopic-guided waterjet. We collected the perioperative data of twenty-eight patients who underwent this procedure for minor and major liver resections between February 2019 and December 2020 from the Magdeburg Registry of minimally invasive liver surgery (MD-MILS). Results: Twenty-eight patients underwent robotic-assisted 3D parenchyma dissection within the investigation period. Twelve cases of major and sixteen cases of minor hepatectomy for malignant and non-malignant cases were performed. Operative time for major liver resections (≥ 3 liver segments) was 381.7 (SD 80.6) min vs. 252.0 (70.4) min for minor resections (*p* < 0.01). Intraoperative measured blood loss was 495.8 (SD 508.8) ml for major and 256.3 (170.2) ml for minor liver resections (*p* = 0.090). The mean postoperative stay was 13.3 (SD 11.1) days for all cases. Liver surgery-related morbidity was 10.7%, no mortalities occurred. We achieved an R0 resection in all malignant cases. Conclusions: The 3D technique for parenchyma dissection in robotic liver surgery is a safe and feasible procedure. This novel method offers an advanced locally controlled preparation of intrahepatic vessels and bile ducts. The combination of precise extrahepatic vessel handling with the 3D technique of parenchyma dissection is a fundamental step forward to the standardization of robotic liver surgery for teaching purposing and the wider adoption of robotic hepatectomy into routine patient care.

## 1. Introduction

With the continued successful implementation of minimally invasive surgery in various surgical specialities including surgical oncology [1,2,3,4,5,6,7,8,9,10,11], notably, robotic surgery has also been accepted for increasing indications [9,10,12,13]. Through continuous development of technical and surgical demands, the indication spectrum has been expanded in general surgery and surgical oncology, involving more complex situations such as major hepatic resections. These can be performed safely with low blood loss, faster postoperative mobilization and less postoperative pain for patients when compared to open procedures while maintaining similar oncologic outcomes [5,6,7,8,9,12,13].

Laparoscopic liver surgery has gained broad acceptance in minor and major liver surgery. Meanwhile, the access to all liver segments is described and even Associating liver partition and portal vein ligation (ALLPS) procedures have been performed [12,13]. It is considered safe and effective with advantages regarding the perioperative measures compared to open operations with similar oncological outcomes [13]. Nevertheless, robotics has some advantages compared to conventional laparoscopy. One big advantage is the so-called EndoWrist which enables the handling of the instrument’s tips in seven degrees of freedom. Furthermore, the stable three-dimensional visualization and the maneuvering of three instruments by the surgeon are steps forward in minimally invasive surgery. These facts make a precise vessel dissection and control during robotic liver resection possible. According to the latest retrospective analysis, the effectiveness of robotic liver surgery is not inferior to open surgery. However, conclusions on several aspects such as operative time, intraoperative blood loss, surgical morbidity, and overall cost-benefit ratio remain controversial [13,14,15,16,17,18,19,20,21,22,23,24].

One challenging step is the parenchymal dissection during robotic liver resection. There is no standardization and various techniques are described so far. Choi et al. report that the parenchymal transection during robotic surgery is the most challenging step because available instruments for parenchymal transection are limited and there is no well-established procedure [5]. Methods such as the “initial traction method”, using suture retraction and parenchyma dissection with monopolar scissors and bipolar forceps as described by Giulianotti et al. [24], the “rubber band suspension method”, as described by Choi et al. [5] and the liver transection technique, using the Harmonic scalpel on the one robotic hand and Maryland forceps on the other [5], have several limitations and cannot be considered as the gold standard.

Facing these challenges, we established a novel, safe and effective technique for the transection of the liver parenchyma during robotic surgery, using three devices simultaneously.

## 2. Materials and Methods

### 2.1. Patients

From February 2019 until December 2020, 12 major and 16 minor liver resections using the “three-device (3D) technique” for liver parenchyma transection were identified from the Magdeburg Registry of Minimally Invasive Liver Surgery (MD-MILS). Robotic liver resections (RLR) using other techniques were excluded from the study. The data were collected prospectively and were analyzed retrospectively. Patient characteristics, perioperative parameters, type of liver resections and liver lesions were evaluated. In a subgroup analysis, minor and major robotic liver resections (removal of ≥ 3 liver segments) were compared. We started our robotic liver program selecting patients without cardiovascular and/or pulmonal co-morbidity, without prior abdominal operations and qualified for minor resection (removal of ≤2 segments). Currently, only patients with liver tumors, which are considered for vascular reconstruction or multivisceral resection, are excluded for robotic liver surgery.

### 2.2. Robotic-Assisted Liver Resection Using the Three Device (3D) Technique

**Figure 1:**
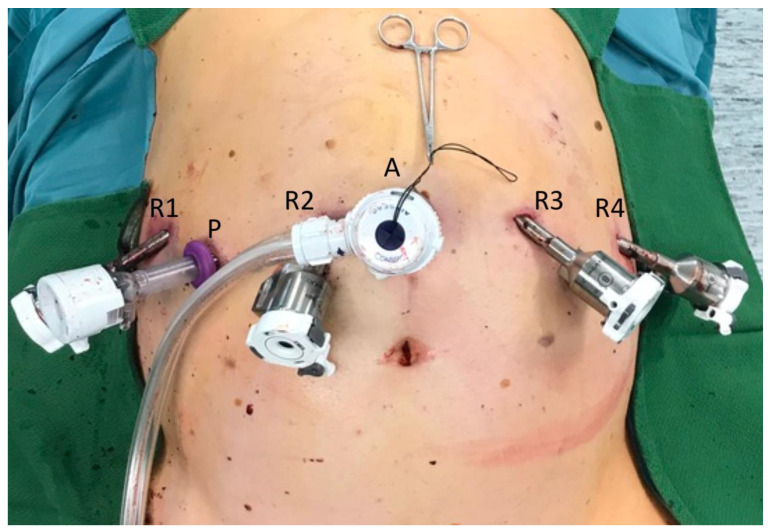
Trocar placement for robotic-assisted liver resection using the three device (3D) method for liver parenchyma dissection (DaVinci Xi System, Intuitive Surgical, Inc., Sunnyvale, CA, USA). R1: Robotic arm 1 used for bipolar Maryland forceps, R2: Robotic arm 2 used for camera, R3: Robotic arm 3 used for monopolar scissors, R4: Robotic arm 4 used for fenestrated grasp. A: Laparoscopic trocar for assistance from the table e.g., driving the waterjet (Erbejet^®^ 2, Erbe Elektromedizin GmbH, Tuebingen, Germany). P: Trocar for pringle maneuver.

**Figure 2:**
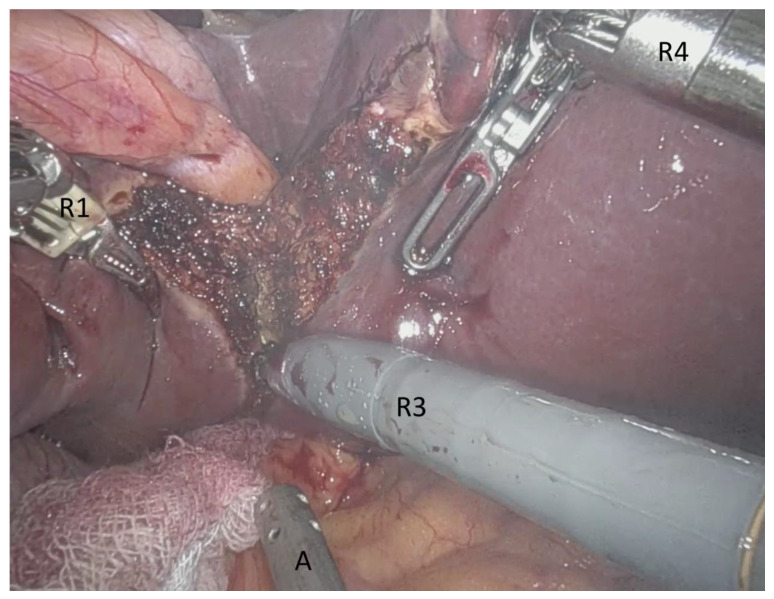
Intraoperative view from the console during robotic-assisted liver resection (DaVinci Xi System, Intuitive Surgical, Inc., Sunnyvale, CA, USA). R1: Robotic arm 1 with bipolar Maryland forceps, A: Laparoscopic instrument (sugger) driven by the assistant from the table, R3: Robotic arm 3 with monopolar scissors, R4: Robotic arm 4 with fenestrated grasps as retraction device.

For RLR we used the Da Vinci Si and X System and since September 2019 the Da Vinci Xi system (Intuitive Surgical, Inc., Sunnyvale, CA, USA). Patients’ and port/trocar placement were performed in a standard fashion, as we described elsewhere [10]:

For the Xi System, the camera port (Robotic arm 2) was positioned right above the umbilicus. Robotic arm 1 was positioned in the right lateral abdomen, robotic arm 3 left, middle-upper abdomen and robotic arm 4 in the left lateral abdomen (Figure 1). A 10-mm trocar was placed at the right side of the patient for the pringle maneuver. On the left or supraumbilical side, we inserted an additional 10-mm trocar for laparoscopic assistance. Robotic arm 4 was mainly used for traction and liver exposure, while via arm 1 and 3 a bipolar forceps (Maryland) and monopolar scissors were placed for tissue preparation (Figure 2).

In cases of major liver resection, we first control the inflow of the liver. This means that major vessels such as the left or right hepatic artery and the left or right main portal branch respective to the liver side resected, are clipped and cut. The left or right bile duct can be cut extra- or intra-hepatically. In cases of left hemi-hepatectomy, the left hepatic vein can be exposed, clipped and cut. The right hepatic vein is usually closed and cut after the end of the parenchyma phase because access to this vessel is more demanding compared to the left side. After in- and outflow are controlled, the liver is mobilized from the inferior vena cava in major and sometimes even in minor liver resections. Notably, small draining veins must also be ligated and divided.

**Figure 3:**
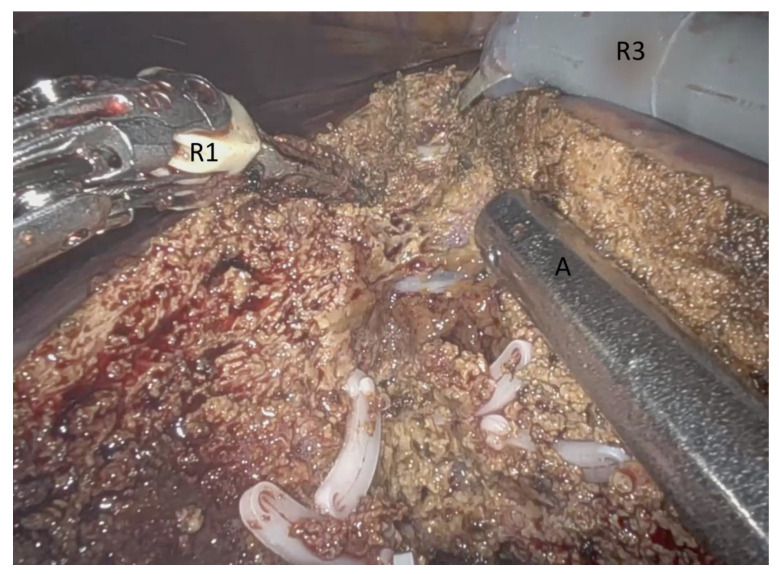
Intraoperative view from the console during robotic-assisted liver resection, using the three device (3D) technique for liver parenchyma dissection (DaVinci Xi System, Intuitive Surgical, Inc., Sunnyvale, CA, USA). R1: Robotic arm 1 with bipolar Maryland forceps, R3: Robotic arm 3 with monopolar scissors, A: Waterjet (Erbejet^®^ 2, Erbe Elektromedizin GmbH, Tuebingen, Germany) driven as laparoscopic instrument via the assistant at the table (Video S1).

After vessel control, the parenchymal dissection begins. The resection margin is marked at the liver surface with the monopolar scissors with ultrasound guidance. The monopolar scissors and the bipolar Maryland forceps are driven by the console surgeon via robotic arm 1 and 3. The Waterjet (Erbejet^®^ 2, Erbe Elektromedizin GmbH, Tuebingen, Germany) is handled by the assistant on the patient table via the 10 mm periumbilical port (Figure 3). This makes the use of three devices (3D) possible for parenchyma dissection. The waterjet elucidates intrahepatic vessels and bile ducts very precisely, carefully and in a controlled fashion. These structures can then be clipped and cut. The bipolar forceps coagulates small vessels and reduces blood loss. For the reason of its fine tip, it can grasp vessels very precisely. The coagulation process of the bipolar forceps is aggravated by the saline of the waterjet. The suction of the waterjet enables clear vision. The monopolar scissors remove liver tissue from the intrahepatic structures and support the efforts of the waterjet. Using these three devices makes the parenchyma phase a fast and straightforward procedure (Video S1).

### 2.3. Definitions

The resection of ≥3 segments was considered as a major resection [25]. The duration of the procedure from the skin incision to the last skin suture was taken as the operation time. We defined the duration of the postoperative hospitalization as length of stay (LOS). The final diagnosis of the liver lesions was determined based on histopathological examination. We defined the posthepatectomy liver failure, intraoperative and postoperative bleeding, bile leak, bile fistula, bilioma, cholangitis, cholangiosepsis, liver abscess and portal vein thrombosis as liver surgery-related complications.

### 2.4. Statistical Analysis

The statistical analysis was performed using IBM SPSS Statistics for Windows, Version 26 (IBM Corp., Armonk, NY, USA). The data were presented using the mean and standard deviation (SD) or the number of cases and percentages in accordance with the type of data. We used Mann–Whitney U test or Pearson’s chi-squared test or Fisher’s exact test for statistical comparison of major and minor groups depending on the type of data.

Statistical significance was considered at a *p*-value of <0.05.

## 3. Results

### 3.1. Patient Demographics and Perioperative Outcomes

In Table 1 patient demographics and perioperative outcomes in patients who underwent minor and major robotic liver surgery using the “three-device (3D) technique” are summarized. The proportion of men and women in our patient cohort was exactly the equal: 14 male (50%) and 14 female (50%) patients. Our patients were 65.3 (SD 11.1) years old on average. The mean operation time was 307.6 (SD 98.4) minutes and mean intraoperative blood loss (IBL) was 358.9 (SD 369.0) ml, respectively. The mean length of postoperative stay was 13.3 (SD 11.1) days in our study.

Three patients (10.7%) in our cohort developed postoperative liver surgery-related complications. One of them suffered from post-hepatectomy hemorrhage after robotic right hemi-hepatectomy due to a cholangiocellular carcinoma (CCA), which needed a revision operation. Bleeding from a side vein of the inferior vena cava was detected intraoperatively, which had been treated successfully. The other two patients had a postoperative bilioma, which was drained percutaneously guided by computed tomography. No patient died during hospitalization.

Sixteen (57.1%) in our cohort underwent abdominal surgery previously and showed significant adhesions.

In our study 12 patients (42.9%) underwent major and 16 patients (57.1%) underwent minor robotic liver resections (Table 1). We performed six right (21.4%) and four left hemi-hepatectomies (14.3%). The other major liver operations were the resection of three liver segments in two cases (21.4%). The minor resections were left lateral liver resections (*n* = 6; 21.4%), anatomical one segment resections (*n* = 4; 14.3%), bisegmentectomies (*n* = 4; 14.3%), atypical resections of one (*n* = 1; 3.6%) and two (*n* = 1; 3.6%) liver segments (Table 2).

### 3.2. Histopathology and Resection Margins

The final diagnosis was determined based on the histopathological examination. This is illustrated in Table 3. Twenty-one patients (75.0%) in our study showed liver malignancies. In all of these cases, we achieved an R0-resection (100.0%). The malignant lesions were colorectal liver metastases in nine cases (32.1%), hepatocellular carcinoma (HCC) in seven cases (25.0%) and cholangiocarcinoma (CCA) in four cases (14.3%). In one case (3.6%), there was detected a mixed tumor of HCC and CCA.

The non-malignant cases were liver hemangioma (*n* = 3; 10.7%), hepatic adenoma (*n* = 2; 7.1%), an inflammatory tumor (*n* = 1; 3.6%) and a bile duct anomaly (*n* = 1; 3.6%).

### 3.3. Comparison of Major and Minor Robotic Liver Resections

In addition, we compared the major and minor RLR regarding perioperative outcomes. As shown in Table 4, the major group showed a significantly longer operation time than the minor group (*p* < 0.01). This was the only significant difference between the major and minor group. There was no significant difference between the groups regarding the length of stay, intraoperative blood loss and liver surgery-related morbidity.

## 4. Discussion

For its perioperative advantages compared to open liver surgery, minimally invasive liver resections have gained increasing acceptance over the past few years. The robot adds technical advantages as already mentioned above which may facilitate advanced procedures. A recently published international consensus statement [13] underlines these technical advantages. In summary, the robotic liver resection was identified as a safe and feasible procedure. The oncologic outcome is comparable to open and conventional laparoscopic procedures [13].

The robot provides ideal instruments for vessel dissection at the in- and outflow of the liver. In addition, the bile duct can be handled safely during RLR. However, according to recent reports and our experiences, the main limitation of robotic liver surgery seems to be the transection of the liver parenchyma. Proposed techniques such as the “Rubber band suspension method” [24] and the liver transection technique, using the harmonic scalpel on the one robotic hand and Maryland forceps on the other or the use of the endo-wristed vessel sealer, have several disadvantages and could not be defined as the gold-standard in terms of robotic liver surgery. The main limitation of harmonic scalpel transection is the loss of the Endowrist function and the remaining risk of serious injury of large intrahepatic vessels in the deep layers of the liver. A recent report underscores the fact that the variety of available instruments in robotic hepatectomy is less than compared to laparoscopic or open techniques [4]. Recent developments, such as an endowristed vessel sealer, have similar limitations as the harmonic scalpel and did not offer a safe, precise parenchyma transection, due to the thickness of its tip [5]. Therefore, other techniques have to be developed to foster robotic liver surgery.

We reported a novel technique of liver parenchyma dissection using three devices simultaneously, which combines the advantages of the available devices for robotic and laparoscopic surgery, following our experience and modifying the applied technique in open procedures with simultaneous use of waterjet and bipolar forceps. Especially and in means of hemostasis, the simultaneous use of the waterjet and bipolar forceps intensifies the bipolar effect through the contact with sodium/chloride solution, used for waterjet. Hereby, intrahepatic smaller vessels can be coagulated sufficiently, and the blood loss is reduced. With the robotic monopolar scissors, the parenchyma dissection is supported which accelerates the procedure. The main advantage of Waterjet in comparison to CUSA, described by others (Lai et al. [26]) is that the coagulation process of the bipolar forceps is aggravated by the saline of the waterjet. Furthermore and through the continuous flow of saline, the instruments remain clean and do not have to be removed in order to remove the necrotic, coagulated tissue from the tip of the robotic instruments.

As far as the limitations and disadvantages of this technique is concerned, we noticed the following points: Firstly, the matter that the bedside assistant has to be skilled and experienced as far as the handling of waterjet is concerned. Our point of view is that there should be a selection for the bedside assistant when performing robotic liver resection using the “3D-technique”. Bedside assistants should already have experience in open procedures, using the waterjet. Furthermore, we firmly believe that console surgeon and bedside assistant should provide a harmonic synchronization during parenchyma dissection. When providing dissection with Waterjet, the instrument has to be in immediate contact with the liver tissue. Otherwise, and when the jet is far away from liver parenchyma there is the disadvantage of water reflection on the camera and consequently of impaired vision and need of repeated removal of camera for cleaning. A further disadvantage of the technique is that this procedure is significantly slower -especially when fibrosis or cirrhosis is present- when compared to parenchyma dissection with other devices. On the other hand, through the very precise dissection of liver parenchyma we prevent significant blood loss during the parenchyma dissection phase. Another limitation of this technique concerns the matter that there is no “Endowrist-function” for Waterjet. That means that there is no flexibility as far as the direction of the waterjet is concerned. According to our experience, we propose to place the assistant-trocar after docking and defining a clear dissection line, in order to avoid problems, such as access limitations to dissection plane—especially in cases for right hemihepatectomy—and loss of flexible movement during dissection.

Based on our experience, the 3D technique seems to offer better local control and a better exposition of the intrahepatic vessels and bile ducts leading to a more precise performance compared to other methods. Furthermore, there is a significant advantage in comparison to laparoscopic procedures through the simultaneous use of three devices.

Although the current evidence for advanced robotic liver surgery is generally weak, we firmly believe that this technique is a step in the right direction for eventual standardization for robotic liver resections. It might lead to improved security and better outcomes and could be one further parameter towards the implementation of more widespread robotic liver surgery. Ultimately, this could enable more patients to benefit from the advantages of this procedure.

Compliance with ethical standards: All procedures performed in studies involving human participants were in accordance with the ethical standards of the University Hospital Magdeburg and with the 1964 Helsinki Declaration and its later amendments or comparable ethical standards.

## Figures and Tables

**Table 1:** Demographics and perioperative outcomes in patients who underwent major and minor robotic liver resection (RLR) using the three device (3D) technique for parenchyma dissection.

		Patients; *n* (SD or Percent)
Total		28 (100.0)
Sex	male	14 (50.0)
	female	14 (50.0)
Age; years		65.3 (11.1)
Operation time; min		307.6 (98.4)
LOS; days		13.3 (11.1)
IBL; mL		358.9 (369.0)
Liver surgery-related morbidity		3 (10.7)
Previous abdominal surgery		16 (57.1)
Dignity of the lesion	malignant	21 (75.0)
	benign	7 (25.0)
R0-resection		21 (100.0)
Major liver resections		12 (42.9)
Minor liver resections		16 (57.1)

IBL = intraoperative blood loss, LOS = length of stay, SD = standard deviation.

**Table 2:** Surgical procedures in patients who underwent major and minor robotic liver resection (RLR) using the three device (3D) technique for parenchyma dissection.

		*n* (%)
Type of liver resection		
	Right hemihepatectomy	6 (21.4)
	Left hemihepatectomy	4 (14.3)
	Left lateral liver resection	6 (21.4)
	Bisegmentectomy	4 (14.3)
	Anatomical one segment resection	4 (14.3)
	Resection of three segments	2 (7.1)
	Atypical two segment resection	1 (3.6)
	Atypical one segment resection	1 (3.6)
Total		28 (100.0)

**Table 3:** Liver tumor pathology in patients who underwent major and minor robotic liver resection (RLR) using the three device (3D) technique for parenchyma dissection.

Type of Liver Lesion		*n* (%)
Malignant lesions		
	Colorectal liver metastases	9 (32.1)
	HCC	7 (25.0)
	CCA	4 (14.3)
	HCC + CCA	1 (3.6)
Benign lesions		
	Liver hemangioma	3 (10.7)
	Hepatic adenoma	2 (7.1)
	Inflammatory tumor	1 (3.6)
	Bile duct anomaly	1 (3.6)
Total		28 (100.0)

CCA = cholangiocellular carcinoma, HCC = hepatocellular carcinoma.

**Table 4:** Perioperative outcomes between major vs. minor robotic liver resections (RLR) using the three device (3D) technique for parenchyma dissection.

	Major Resections *n* (% or SD)	Minor Resections *n* (% or SD)	*p*-Value
Total	12 (42.9)	16 (57.1)	
Operation time; min	381.7 (80.6)	252.0 (70.4)	<0.01
LOS; days	15.8 (11.6)	11.3 (10.7)	0.159
IBL; mL	495.8 (508.8)	256.3 (170.2)	0.090
Liver surgery related morbidity	3 (25.0)	0 (0.0)	0.067

IBL = intraoperative blood loss, LOS = length of stay, SD = standard deviation.

## Data Availability

The data presented in this study are available on request from the corresponding author. The data are not publicly available due to restrictions e.g., privacy or ethical.

## Supplementary Materials

The following are available online at https://zenodo.org/record/5573683#.YWqcqLjP1aQ, Video S1: Three device parenchyma dissection in robotic surgery.
